# PgRNA kinetics predict HBsAg reduction in pregnant chronic hepatitis B carriers after treatment cessation

**DOI:** 10.3389/fcimb.2022.1055774

**Published:** 2022-12-12

**Authors:** Chun-Rui Wang, Xiao-qin Liu, Hu Li, Qian Zhang, Guo-Chao Zhong, Qiao Tang, Yunan Chang, Jin-Song Wang, Yuan-qin Duan, Peng Hu

**Affiliations:** ^1^ Department of Infectious Diseases, Institute for Viral Hepatitis, The Key Laboratory of Molecular Biology for Infectious Diseases, Chinese Ministry of Education, The Second Affiliated Hospital of Chongqing Medical University, Chongqing, China; ^2^ Department of Hepatobiliary Surgery, The Second Affiliated Hospital of Chongqing Medical University, Chongqing, China

**Keywords:** novel biomarkers, prediction model, NAs prophylaxis, mother-to-child transmission (MTCT), pregnance

## Abstract

**Background:**

Pregenomic RNA (pgRNA) and hepatitis B core-related antigen (HBcrAg) play significant roles in predicting discontinuing treatment outcomes. However, their role in pregnancy has rarely been reported. We aimed to evaluate the performance of pgRNA and HBcrAg kinetics in predicting HBeAg seroconversion and HBsAg reduction postpartum in HBeAg-positive pregnant women.

**Methods:**

Pregnant HBeAg-positive patients receiving antiviral prophylaxis and ceasing treatment postpartum were included. PgRNA and HBcrAg levels were measured before treatment, at 32 weeks of gestation, and at treatment withdrawal postpartum. Other virological and biochemical parameters were regularly examined until 96 weeks postpartum.

**Results:**

Of 76 pregnant chronic hepatitis B (CHB) carriers with a median treatment duration of 18.1 weeks, HBeAg seroconversion and HBsAg reduction >0.3 log_10_ IU/mL at 96 weeks postpartum occurred in 8 (10.5%) and 13 (17.1%) patients, respectively. HBsAg correlated most strongly with pgRNA, while HBeAg correlated most strongly with HBcrAg. Multivariable regression analysis revealed that postpartum pgRNA decline and peak ALT levels were independent predictors of HBsAg reduction. The area under the curve of the regression model was 0.79 and reached as high as 0.76 through bootstrapping validation. The calibration plot showed that the nomogram had a performance similar to that of the ideal model. A decision tree was established to facilitate application of the nomogram. In addition, HBcrAg kinetics, as an independent predictor, performed poorly in predicting HBeAg seroconversion.

**Conclusions:**

Postpartum pgRNA decline together with peak ALT levels may identify patients with a higher probability of HBsAg reduction after treatment cessation postpartum among pregnant CHB carriers receiving antiviral prophylaxis.

## Introduction

Chronic hepatitis B (CHB) is a serious public health problem, resulting in approximately 800,000 deaths every year (Liu et al., 2019). China has the largest burden of hepatitis B virus (HBV) infection worldwide (The Polaris Observatory Collaborators, 2018). In China, approximately 6% of women giving birth live with HBV, among whom the hepatitis B e antigen (HBeAg)-positive rate is up to 30% (Jing et al., 2020). Mother-to-child transmission is the primary route of HBV transmission in China. Thus, antiviral prophylaxis in pregnant CHB patients is necessary in preventing HBV transmission (Zhou et al., 2020).

During pregnancy, the maternal immune system tolerates fetal antigens by suppressing cell-mediated immunity while retaining normal humoral immunity (Gaunt and Ramin, 2001). After delivery, these adaptations disappear and the immune system reconstructs, which could influence liver disease activity (ter Borg et al., 2008). The reported prevalence of alanine aminotransferase (ALT) flares after cessation postpartum varies from 5% to 62% (ter Borg et al., 2008; Xu et al., 2009; Han et al., 2011; Liu et al., 2013; Pan et al., 2016; Liu et al., 2017; Chang et al., 2018), potentially related to the flare definition, patient characteristics, or occurrence of virological rebound. It has been hypothesized that the rapid reactivation of the immune system against HBV antigens is responsible for postpartum ALT flares (ter Borg et al., 2008). Choi et al. (2021) concluded that ALT flares during PEG-IFN-α treatment were associated with subsequent HBsAg and HBV RNA decline, and predicted subsequent HBsAg loss. Serum pregenomic RNA (pgRNA) and hepatitis B core-related antigen (HBcrAg) are potential surrogate markers for covalently closed circular DNA (cccDNA) transcriptional activity (Wang et al., 2016). Currently, only three studies have analyzed pgRNA and HBcrAg in HBV-infected pregnant women (Zhang et al., 2018; Patel et al., 2019; Wang et al., 2022). However, the roles of pgRNA and HBcrAg levels in predicting long-term outcomes following treatment cessation postpartum have not been investigated. Hence, we aimed to investigate the performance of pgRNA and HBcrAg kinetics in predicting HBeAg seroconversion and HBsAg reduction in HBeAg-positive pregnant CHB carriers.

## Materials and methods

This study strictly conformed to the Ethical Guidelines of the 1975 Declaration of Helsinki and was approved by the Ethics Committee of the Second Affiliated Hospital of Chongqing Medical University. Written informed consent was obtained from all the subjects. This study was registered with the Chinese Clinical Trial Registry (ChiCTR2100054116).

### Study population

The study population comprised pregnant women with HBV infection who visited the Second Affiliated Hospital of Chongqing Medical University outpatient clinic from January 2019 to September 2021. HBeAg-positive pregnant CHB carriers who received antiviral prophylaxis at 24–28 weeks of gestation and ceased treatment at 4–8 weeks postpartum were also included. Patients were excluded if they received treatment before pregnancy or were coinfected with other hepatotropic virus infections, HIV infection, or were complicated with cirrhosis and pregnancy-related diseases.

### Study setting

According to the 2019 Chinese Guidelines for Prevention and Treatment of CHB (Wang and Duan, 2021), antiviral prophylaxis was performed at 24–28 weeks of gestation among pregnant CHB patients with HBV DNA levels >200,000 IU/mL and ceased within 12 weeks postpartum. Patients with drug withdrawal were regularly followed-up at 1- or 3-month intervals for an additional 2 years to analyze 96 weeks postpartum outcomes, which included HBeAg seroconversion (defined as the loss of serum HBeAg and the development of anti-HBe antibodies during follow-up (EASL, 2017)) and HBsAg reduction (defined as HBsAg decrease >0.3 log_10_ IU/mL from baseline to the end of follow-up) (Zhang et al., 2020; Janssen et al., 2020).

### Clinical and laboratory data collection

Serum samples were collected at 24–28 weeks of gestation (referred to as baseline), 32–36 weeks of gestation (referred to as near delivery), and treatment withdrawal postpartum (referred to as postpartum) for serum pgRNA and HBcrAg measurements. Serum pgRNA was measured using an ABI7500 quantitative real-time polymerase chain reaction (PCR) system (ABI Laboratories, USA) , with a detection range of 2×10 (The Polaris Observatory Collaborators, 2018) to 1×10 (Pan et al., 2016) copies/mL. Serum HBcrAg was measured using a chemiluminescent immunoassay, Lumipulse G1200 automated analyzer (Fujirebio, Tokyo, Japan), with a sensitivity of 2 log U/mL. Demographic and clinical data, including age, parity status, infant sex, antiviral therapy regimen, complete blood count, liver function test, and classic HBV markers, were collected during pregnancy and postpartum.

### Statistical analysis

Five major statistical analyses were performed. First, a descriptive analysis was performed. Fisher’s exact test and Mann–Whitney U test were conducted for categorical and continuous variables between groups, respectively. Spearman’s correlation test was used to evaluate the correlations between continuous biomarkers. Second, multivariable logistic regression was used to identify independent factors associated with postpartum outcomes. Of note, baseline variables that were considered clinically relevant or showed a statistically significant association with the outcome of interest in the univariable logistic regression model were entered into the multivariable logistic regression model. Third, a nomogram was constructed based on the screened independent risk factors to predict the probability of an outcome of interest. Fourth, we further calculated the discrimination and calibration of this nomogram indicated by the area under the curve (AUC) and calibration curve, respectively, which were validated by 1000 bootstrap resamplings. Finally, by calculating the appropriate nodes and complex parameters, a decision tree was established to provide a simple decision-making process for clinicians. All data analyses were conducted using the SPSS software (version 26.0) and R software (version 3.6.1). The statistical significance level was set at *P*<0.05, using a two-tailed test.

## Results

### Characteristics of included patients

Of 152 HBV-infected pregnant women, 76 were included, with a median treatment duration of 18.1 weeks (range 14.6–20.7 weeks) (**Figure S1**). The demographic and clinical characteristics of the included patients are shown in **Table 1**. HBeAg seroconversion and HBsAg reduction at 96 weeks postpartum were observed in 8 (10.5%) and 13 (17.1%) patients, respectively. Compared with those in the non-seroconversion group, patients in the seroconversion group had increased ALT levels [56.5 (IQR 37.0–65.2) vs. 20.0 (IQR 15.8–28.2) U/L, *P*<0.01], decreased HBeAg [2.4 (IQR 1.7–3.1) vs. 3.2 (IQR 3.0–3.3) log_10_PEIU/mL, *P*=0.02], and HBsAg [4.3 (IQR 4.0–4.4) vs. 4.5 (IQR 4.3–4.7) log_10_IU/mL, *P*=0.05]. No significant differences were noted in HBV DNA, pgRNA, or HBcrAg levels between the two groups (all *P*>0.05). Compared with those in the non-reduction group, patients in the HBsAg reduction group had increased ALT levels [28.0 (IQR 19.0–38.0) vs. 20.0 (IQR 15.5–31.0) U/L, *P*=0.01] and increased peak ALT values postpartum (hereafter referred to as postpartum ALT_max_) [67.0 (IQR 46.0–102.0) vs. 40.0 (IQR 27.0–83.6) U/L, *P*=0.04]. We further plotted changing patterns of HBsAg and ALT levels for each patient with HBsAg reduction (N=13) during follow-up (**Figure S2**) and found that peak ALT levels postpartum >40 U/L were observed in 10 (76.9%) patients. No significant differences were noted in other host or viral biomarkers between the two groups.

**Table 1:** Demographics and clinical characteristics of 76 pregnant CHB carriers with HBeAg positive at baseline.

	All patients	Without HBeAg seroconversion	HBeAg seroconversion	*P* value	Without HBsAg reduction N=63	HBsAg reduction N=13	*P* value
	N=76	N=68	N=8				
Age, years	28.0 (26.0-31.0)	28.0 (26.0-31.0)	26.5 (25.5-27.2)	0.06	28.0 (26.0-31.0)	26.0 (25.0-30.0)	0.60
Parity status				0.47			0.16
The first pregnancy	71 (93.4%)	64 (94.1%)	7 (87.5%)		60 (95.2%)	11 (84.6%)	
The second pregnancy	5 (6.6%)	4 (5.9%)	1 (12.5%)		3 (4.8%)	2 (15.4%)	
Male Infant, n (%)	34 (44.7%)	31 (45.6%)	3 (37.5%)	0.66	28 (44.4%)	6 (46.2%)	0.91
Antiviral prophylaxis TDF vs. LDT	33 (43.4%) vs. 43 (56.6%)	30 (44.1%) vs.38 (55.9%)	3 (37.5%) vs.5 (62.5%)	0.72	29 (46.0%) vs. 34 (54.0%)	4 (30.8%) vs. 9 (69.2%)	0.31
Treatment duration, weeks	18.1 (14.6-20.7)	18.3(14.7-20.9)	15.4 (12.4-17.4)	0.31	17.4 (14.2-21.1)	18.7 (16.4-20.0)	0.62
ALT, U/L	21.5 (16.0-32.0)	20.0 (15.8-28.2)	56.5 (37.0-65.2)	<0.01	20.0 (15.5-31.0)	28.0 (19.0-38.0)	0.01
Postpartum ALT _max_	46.5 (30.2-87.0)	46.0 (28.0-85.5)	55.0 (39.0-129.8)	0.08	40.0 (27.0-83.6)	67.0 (46.0-102.0)	0.04
HBV DNA, log_10_IU/mL	6.9 (6.6-7.4)	7.0 (6.6-7.4)	6.5 (5.7-6.9)	0.10	6.8 (6.5-7.3)	7.3 (6.8-7.9)	0.06
HBsAg, log_10_IU/mL	4.5 (4.3-4.6)	4.5 (4.3-4.7)	4.3 (4.0-4.4)	0.05	4.5 (4.2-4.6)	4.7 (4.4-5.0)	0.06
HBeAg, log_10_PEIU/mL	3.2 (2.9-3.3)	3.2 (3.0-3.3)	2.4 (1.7-3.1)	0.02	3.2 (2.9-3.3)	3.2 (3.0-3.3)	0.89
HBcrAg, log_10_U/mL	8.6 (8.4-8.7)	8.6 (8.4-8.7)	8.6 (8.2-8.6)	0.83	8.6 (8.4-8.7)	8.7 (8.6-8.8)	0.43
PgRNA, log_10_copies/mL	7.8 (7.6-8.1)	7.8 (7.7-8.1)	7.6 (7.1-8.4)	0.96	7.8 (7.6-8.0)	8.1 (7.9-8.6)	0.21

Continuous variables were expressed as median [interquartile range (IQR)], and categorical variables were expressed as counts (percentage). Baseline: at 26 ± 2 weeks of gestation; TDF, tenofovir disoproxil fumarate; LDT, telbivudine; HBV, hepatitis B virus; DNA, deoxyribonucleic acid; ALT, alanine aminotransferase; postpartum ALT_max_, peak ALT level postpartum; HBsAg, hepatitis B surface antigen; HBeAg, hepatitis B e antigen; pgRNA, pregenomic RNA; HBcrAg, hepatitis B core-related antigen. HBsAg reduction, HBsAg decrease >0.3log_10_IU/mL from baseline to last date of follow-up.

**Figure 1:**
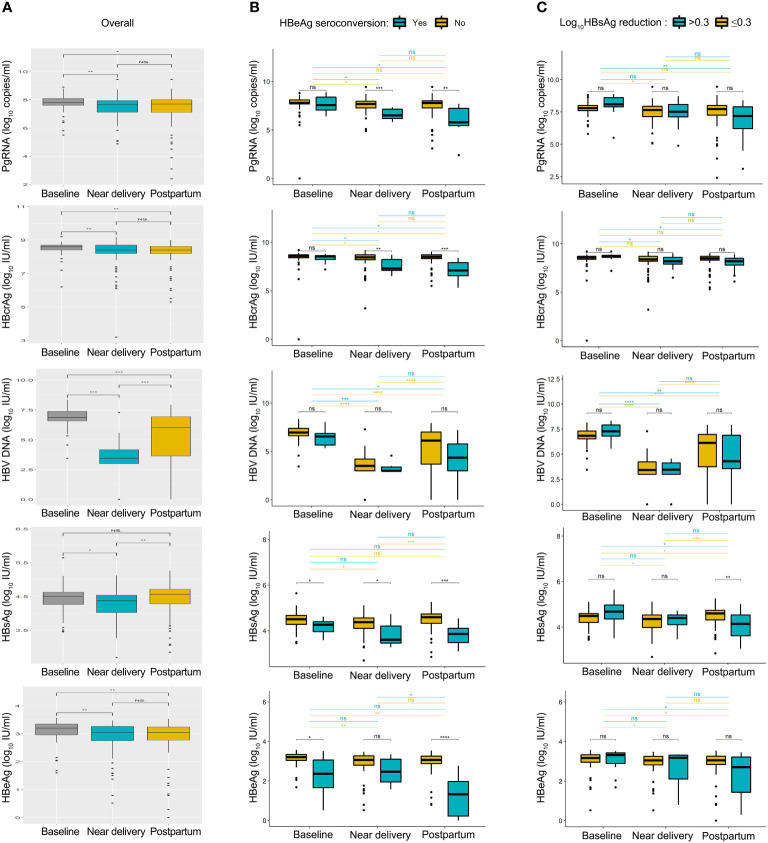
Summary of HBV biomarkers from baseline to postpartum. Kinetics of pgRNA, HBcrAg, HBV DNA, HBsAg and HBeAg levels was shown in all patients **(A)**, patients with or without HBeAg seroconversion **(B)**, patients with or without HBsAg reduction **(C)**. Baseline, 24-28 weeks of gestation; near delivery, 32-36 weeks of gestation; postpartum, at 2-6 weeks after delivery. NS = non-significant (p > 0.05), ^*^ *P* < 0.05, ^**^ *P* < 0.01, ^***^ *P* < 0.001, ****P < 0.0001.

The kinetics of HBV biomarkers are summarized in **Figure 1**. HBV biomarkers in the five groups changed dynamically during pregnancy and postpartum in different patterns. The kinetics of HBcrAg and HBV DNA levels showed similar changing patterns between patients with and without HBeAg seroconversion. PgRNA decreased significantly postpartum in the seroconversion group, but not in the non-seroconversion group; HBsAg and HBeAg decreased significantly near delivery in the seroconversion group but not in the non-seroconversion group. The kinetics of HBcrAg and HBV DNA levels showed similar changing patterns between patients with or without HBsAg reduction; kinetics of HBsAg and HBeAg levels showed similar changing patterns between the two groups; pgRNA decreased significantly postpartum in the HBsAg reduction group but not in the non-reduction group.

No significant differences were found between tenofovir disoproxil fumarate and telbivudine groups in terms of HBeAg seroconversion rate [9.1% (3/33) vs. 11.6% (5/43), *P*=0.72] and HBsAg reduction [12.1% (4/33) vs. 20.9% (9/43), *P*=0.31] (**Table S1**). In addition, neither maternal (age, parity status, infant sex, alanine transaminase) nor viral biomarkers (pgRNA, HBcrAg, HBV DNA, HBsAg, and HBeAg) showed any significant difference between the tenofovir disoproxil fumarate and telbivudine groups (all *P*>0.05).

### Serum HBsAg correlated most strongly with pgRNA levels during pregnancy and postpartum

We analyzed the overall correlation between serum pgRNA and HBcrAg levels and other HBV markers (**Figure S3**). Overall, HBsAg levels were found to be most strongly related to pgRNA levels (r=0.59, P<0.01) and showed a slightly weaker association with HBcrAg levels, whereas serum HBeAg was correlated most strongly with HBcrAg (r=0.60, *P*<0.01), and showed a weaker association with pgRNA (r=0.41, *P*<0.01). Significant correlations between the above biomarkers were also observed during pregnancy and postpartum period.

### Decreased pgRNA and HBcrAg levels in HBeAg seroconversion and HBsAg reduction groups

**Figure 2:**
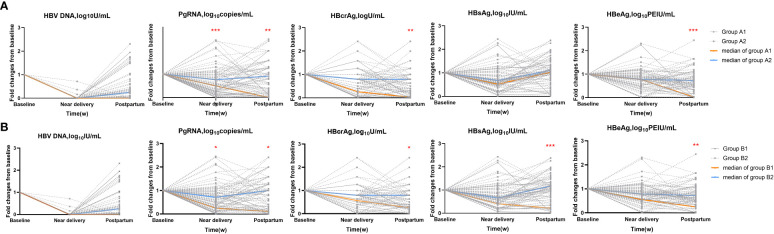
Fold changes of HBV markers in subgroups. **(A)** HBeAg seroconversion group (Group A1) and non-seroconversion group (Group A2). **(B)** HBsAg reduction (Group B1) and non-reduction group (Group B2). *=p < 0.05, **= p < 0.01, ***= p < 0.001.

**Figure 3:**
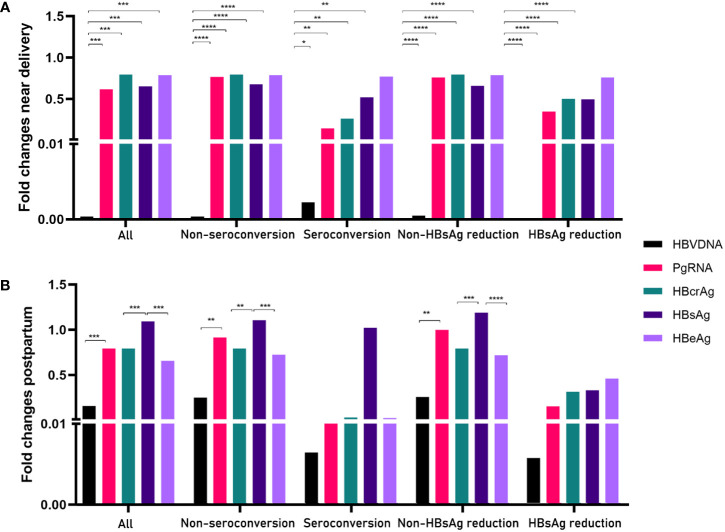
Comparisons of fold changes near delivery **(A)** and postpartum **(B)** in five groups. *= p < 0.05, **= p < 0.01, ***= p < 0.001, ****= p < 0.0001.

Next, we calculated the fold changes of HBV markers in the HBeAg seroconversion and HBsAg reduction groups **(** **Figure 2** **).** Serum PgRNA and HBcrAg levels decreased more rapidly in patients with HBeAg seroconversion than in those without; a similar decreased pattern was observed between the HBsAg reduction and non-reduction groups. No significant differences were noted in the fold changes of HBV DNA levels between the HBeAg seroconversion and non-seroconversion groups (*P*>0.05), or between the HBsAg reduction and non-reduction groups (*P*>0.05). We then compared the fold changes in HBV markers near delivery and postpartum in the five groups (**Figure 3** **)**. Similar trends were noted in the comparisons of fold changes near delivery among the five groups. We observed significant differences in the fold changes near delivery between HBV DNA and pgRNA, HBcrAg, HBsAg, and HBeAg in the five groups (all *P*<0.05). During postpartum, we found significant differences in fold changes between HBV DNA and pgRNA, between HBcrAg and HBsAg, and between HBsAg and HBeAg in the non-seroconversion and non-HBsAg reduction groups (all *P*<0.01), whereas these differences were not found in the HBeAg seroconversion and HBsAg reduction groups (all *P*>0.05).

### PgRNA kinetics independently predicted HBsAg reduction postpartum

**Figure 4:**
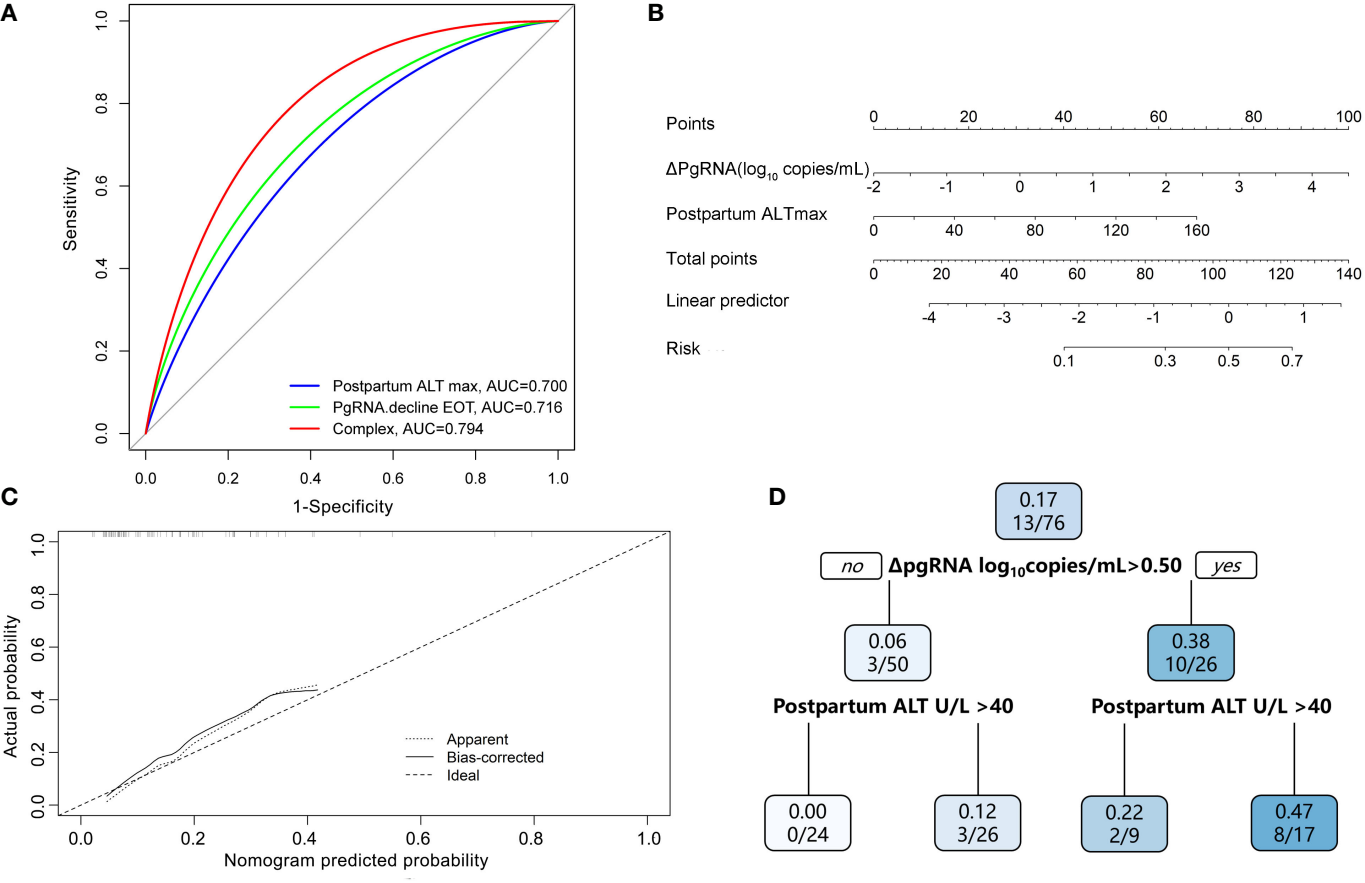
Predictive model of HBsAg reduction. **(A)** AUC performance of ΔpgRNA at the end of treatment (EOT), Postpartum ALT _max_ and their combinations. **(B)** Calibration curve for the combined prediction model to predict probability of HBsAg reduction. **(C)** Nomogram to predict probability of HBsAg reduction in pregnant CHB carriers with HBeAg positive. **(D)** A decision tree for the HBsAg reduction outcome predictive model. PgRNA decline EOT means decline from baseline to postpartum; Postpartum ALT _max_ means peak ALT level postpartum.

Multivariable logistic regression analyses revealed that the pgRNA decline from baseline to postpartum (ΔpgRNA) and postpartum ALT_max_ were independent predictors of HBsAg reduction (**Table 2**). The AUC value was calculated to estimate the advantages of ΔpgRNA, postpartum ALT_max_, and their combined performance (**Figure 4A** **)**. The AUC of the combined biomarkers was 0.79 (95% confidence interval (CI), 0.67–0.92] and reached as high as 0.76 *via* bootstrapping validation (**Table S2** **)**. In addition, we found that ALT levels at baseline and HBcrAg decline from baseline to postpartum (ΔHBcrAg) were independent predictors of HBeAg seroconversion. The AUC value was calculated to estimate the advantage of ΔHBcrAg, ALT at baseline, and their combined performance (**Table S3** **)**. We further calculated the calibration of the combined model consisting of baseline ALT and ΔHBcrAg. The prediction model of ΔHBcrAg plus ALT at baseline showed poor calibration performance, although it had a high AUC value (0.99) (**Figure S4** **).**

**Table 2:** Logistic regression analysis for factors of HBeAg seroconversion and HBsAg reduction postpartum in HBeAg positive carriers (N=76).

Variables^*^	HBeAg seroconversion				HBsAg decrease>0.3logIU/mL			
	Univariate analysis		Multivariate analysis		Univariate analysis		Multivariate analysis	
	OR (95% CI)	*P* Value	OR (95% CI)	*P* Value	OR (95% CI)	*P* Value	OR (95% CI)	*P* Value
Age, year	0.81 (0.65-1.02)	0.07			0.96 (0.82-1.12)	0.59		
Infant gender	0.72 (0.16-3.24)	0.66			1.07 (0.32-3.55)	0.91		
Type of therapy	1.32 (0.29-5.95)	0.22			1.92 (0.54-6.89)	0.32		
Pregnancy status	2.29 (0.22-23.44)	0.49			3.64 (0.54-24.34)	0.18		
HBVDNA, log_10_IU/mL	0.54 (0.25-1.17)	0.12			2.54 (0.96-6.67)	0.06		
ALT, U/L	1.14 (1.06-1.23)	0.00	1.18(1.05-1.32)	0.01	1.04 (1.00-1.07)	0.03		
HBsAg, log_10_IU/mL	0.18 (0.03-1.07)	0.06			5.46 (0.92-32.54)	0.06		
HBeAg, log_10_PEIU/mL	0.19 (0.05-0.71)	0.01			0.87 (0.23-3.26)	0.84		
PgRNA, log_10_copies/mL	0.72 (0.24-2.16)	0.57			2.76 (0.76-9.97)	0.12		
HBcrAg, log_10_U/mL	0.45 (0.13-1.64)	0.23			2.35 (0.36-15.29)	0.37		
ΔHBVDNA, log_10_IU/mL	1.18 (0.82-1.69)	0.35			1.37 (1.02-1.86)	0.04		
ΔPgRNA.log_10_copies/mL	2.51 (1.36-4.63)	0.00			1.90 (1.14-3.17)	0.01	2.00(1.17-3.45)	0.01
ΔHBcrAg.log_10_U/mL	4.99 (1.88-13.24)	0.00	6.5(1.41-29.87)	0.02	2.17 (0.99-4.72)	0.05		
ΔHBsAg, log_10_IU/mL	5.62 (1.27-24.92)	0.02			74.04 (7.68-713.57)	0.00		
ΔHBeAg, log_10_PEIU/mL	7.76 (2.54-23.78)	0.00			2.10 (0.95-4.60)	0.06		
Postpartum ALT _max_	1.02 (0.99-1.04)	0.09			1.02 (1.00-1.03)	0.04	1.02(1.00-1.04)	0.04

^*^The variables enrolled in the logistic regression analysis were age, HBVDNA, ALT, HBsAg, HBeAg, pgRNA, HBcrAg level, ΔHBVDNA, ΔpgRNA, ΔHBcrAg, ΔHBsAg, ΔHBeAg (continuous variable), infant gender (male vs. female), type of therapy (TDF vs. LDT), pregnancy status (first vs. second pregnancy). Δ means variable decline from baseline to postpartum; Postpartum ALT _max_ means peak ALT level postpartum.

### ΔpgRNA plus postpartum ALT_max_ improved the estimation of HBsAg reduction postpartum

Based on the results of the multivariate logistic regression analyses, we constructed a nomogram by combining predictive factors, including postpartum ALT_max_ and ΔpgRNA, to facilitate the prediction of HBsAg reduction postpartum after treatment cessation in pregnant CHB carriers (**Figure 4B** **).** Calibration curves and decision trees were constructed to estimate the clinical utility of the nomogram. The calibration curve showed that the performance of our nomogram was similar to that of an ideal model (**Figure 4C**). The decision tree showed that the combined biomarkers had a superior performance in predicting the probability of postpartum HBsAg reduction (**Figure 4D**). When ΔpgRNA >0.50 log_10_ copies/mL plus postpartum ALT_max_ >40 U/L, the possibility of HBsAg reduction >0.3 log_10_ IU/mL reached as high as 47%.

## Discussion

Currently, only three studies have analyzed pgRNA or HBV RNA levels in HBV-infected pregnant patients (Zhang et al., 2018; Patel et al., 2019; Wang et al., 2022). One prospective study in 46 pregnant CHB patients described HBV total RNA and pgRNA levels during pregnancy and postpartum and their possible associations with established HBV viral markers. Another retrospective study showed that genotype had a certain influence on HBV RNA kinetics following NA therapy. The degree of decline in HBV RNA in patients with genotype B was significantly higher than that in patients with genotype C. In addition, an observational cohort study analyzed the relationship between HBV DNA, HBV RNA, and HBsAg and the predictive value for mother-to-child transmission. The role of HBcrAg levels in pregnant CHB carriers has rarely been reported. It is well known that the aim of nucleos(t)ide analog (NA) therapy is to maintain viral suppression during treatment (EASL, 2017). HBsAg loss is regarded as an effective treatment endpoint, termed “functional cure”; however, it is rarely achieved with current antiviral regimes (Cornberg and Höner Zu Siederdissen, 2014). In our study, after analyzing 76 pregnant CHB carriers with a median treatment duration of 18.1 weeks, we found that HBsAg reduction >0.3 log_10_ IU/mL at 96 weeks postpartum occurred in 13 (17.1%) patients and concluded that pgRNA was not only positively correlated with classic viral biomarkers but also acted as an independent predictor of HBsAg reduction. The combination of pgRNA decline postpartum and postpartum ALT_max_ performed satisfactorily in predicting HBsAg reduction after treatment cessation postpartum.

By estimating the overall correlation between HBV markers, we found the strongest correlation between serum pgRNA and HBcrAg (r=0.630, *P*<0.001) and a slightly weaker correlation between serum pgRNA and HBsAg or HBV DNA (r=0.590 and r=0.480, respectively, all *P*<0.001). Consistent with a previous study, Wang et al. (2021) reported that in HBeAg-positive patients, serum HBV RNA was weakly correlated with HBV DNA (r=0.449, P<0.001) but moderately correlated with HBsAg (r=0.557, P<0.001) and HBcrAg (r=0.601, P<0.001). In another Hong Kong study, Mak et al. (2021) concluded serum HBV RNA correlates best with HBcrAg in HBeAg-positive NA-treated CHB patients (r=0.795, P<0.001), and to a lesser extent with serum HBsAg (r=0.719, P <0.001) or HBV DNA (r=593, P<0.001). Both pgRNA and HBcrAg originated only from cccDNA, which may explain the strong correlation between them. We also found that the correlation between serum HBsAg and pgRNA (r=0.59, P<0.01) was the highest, but weakened between HBsAg and HBcrAg (r=0.53, P<0.001) and HBV DNA (r=0.40, P<0.001). The correlation between serum HBeAg and HBcrAg (r=0.60, P<0.01) was the highest, but weakened between HBeAg and pgRNA (r=0.41, P<0.001) or HBV DNA (r=0.39, P<0.001). Similar to a previous Hong Kong study (Mak et al., 2021), they showed good correlation between HBsAg and pgRNA (r=0.719, *P <*0.001) and weaker correlation between HBsAg and HBcrAg (r=0.632, *P*<0.001) and HBV DNA (r=0.547, *P*<0.001). It is well known that HBcrAg combines the antigenic reactivity resulting from denatured HBeAg, HBcAg, and core-related protein (Mak et al., 2018), which may explain the strong correlation between serum HBeAg and HBcrAg.

Our subsequent analysis also supports this result. Multivariable regression analyses revealed that ΔpgRNA postpartum levels were independently and positively correlated with HBsAg reduction. Several studies have reported that HBV RNA decline is associated with higher rates of off-treatment sustained response among patients treated with NAs or peginterferon alfa (PEG-IFN) (Huang et al., 2015; van Bömmel et al., 2018; Luo et al., 2020; van Campenhout et al., 2020). Janssen et al. (van Campenhout et al., 2020) investigated whether HBV RNA can predict serological response to peginterferon treatment and revealed that at week 12, a trend was observed toward lower HBV RNA levels in patients with HBsAg loss (3.8 vs. 4.9 log copies/mL, *P*=0.11) and more HBV RNA decline from baseline (−3.2 vs. −2.0 log copies/mL, *P*=0.06). Another study (Huang et al., 2015) reported that on-treatment low-serum HBV RNA levels at treatment week 12 (adjusted hazard ratio=0.908, 95% CI 0.829, 0.993, *P*=0.035) independently predicted the initial virological response in NA-treated patients with CHB. A previous American study (van Bömmel et al., 2018) demonstrated that at week 12, an HBV RNA cutoff of 5.5−log10 copies/mL identified a higher proportion of non-responders to PegIFN alfa-2a treatment (30%) than an HBV DNA cutoff of 8.9−log10 IU/mL (22%) or HBeAg cutoff of 2.7−log10 IU/mL (29%). Together with our results, these results indicate that HBV pgRNA may not only correlate well with classic viral biomarkers but also act as an independent predictor of HBsAg reduction.

Another independent predictor, postpartum ALT elevation, is associated with subsequent HBsAg decline. ALT flares are postulated to be primarily immune-mediated and may be beneficial for successful clearance of infection. The postpartum period appears to have an effect on HBeAg seroconversion, with several small studies showing higher than expected seroconversion rates in the early postpartum period (12% of 40 pregnancies (Lin et al., 2006); 17% of 30 women (Lin et al., 1989); in the Melbourne cohort study (Giles et al., 2015), 7% of 30). In addition, on-treatment flares have been associated with declines in serum HBV DNA and HBsAg (Flink et al., 2005; Sonneveld et al., 2013). Wong et al. (2018) reported that ALT flares are independently associated with HBsAg loss. Another study (Jeng et al., 2016) showed that early HBsAg reduction increased in an ALT (<5, 5–10, 10–20, and ≥20×ULN, *P*=0.001) level-dependent manner. In our study, peak ALT level postpartum was higher in subjects who achieved HBsAg reduction and independently associated with HBsAg response, possibly associated with a stronger antiviral effect. The characteristics of T-cell immunity were distinct between mothers with postpartum ALT flare and those without (Huang et al., 2021). T cells in mothers with ALT flares produced more pro-inflammatory cytokines (IFN-γ, IL-21, TNF-α, and IL-2) or less anti-inflammatory cytokines (IL-10) than those in mothers without ALT flares. Next, we constructed a nomogram consisting of peak ALT levels and ΔpgRNA postpartum with satisfactory AUC values and calibration performance. As the decision tree shown, the incidence of HBsAg reduction was significantly higher in patients with postpartum ΔpgRNA >0.50 log_10_ copies/mL plus peak ALT level >40 U/L than in those with postpartum ΔpgRNA <0.50 log10 copies/mL or peak ALT level <40 U/L.

Notably, multivariate regression analysis revealed that ALT and ΔHBcrAg were independent factors for HBeAg seroconversion. However, the univariate model that included baseline ALT >30 U/L had an AUC value of 0.95. Hence, the contribution of ΔHBcrAg was marginal. The prediction model of ΔHBcrAg plus ALT at baseline showed an unsatisfactory calibration performance. Thus, we did not develop a nomogram or decision tree for the HBeAg seroconversion model. More detailed kinetic studies with larger sample sizes are needed to characterize pgRNA and HBcrAg kinetics under NA treatment among pregnant HBV carriers, which will help us to understand HBV-host interactions and the NA mode of action.

To the best of our knowledge, this is the first study to evaluate the performance of serum pgRNA and HBcrAg levels in predicting off-treatment HBeAg seroconversion and HBsAg decline in well-characterized cohorts of pregnant CHB carriers with close monitoring and comprehensive off-treatment data collection in China. Nonetheless, our study has several limitations. First, it was a retrospective study with a limited number of patients. Therefore, our results should be interpreted with caution. Prospective analyses are required to validate our findings in a large and diverse population. Second, because of the limited number of patients with HBsAg loss (1/76) in our postpartum 96-week cohort, we could not further assess the prediction of pgRNA and HBcrAg levels for HBsAg loss following treatment. Third, the study population included pregnant CHB carriers in the immune tolerance stage receiving antiviral prophylaxis. Therefore, the conclusions of this study cannot be generalized to all patients with HBV infection. More studies that include patients with different infection periods or patients with low viral loads are needed to verify the roles of pgRNA and HBcrAg in predicting HBeAg seroconversion and HBsAg reduction.

In conclusion, postpartum pgRNA decline together with peak ALT level, as independent predictors, were positively correlated with the rates of HBsAg reduction postpartum among pregnant HBeAg-positive CHB carriers, indicating that pgRNA could be a potential serological marker for monitoring HBV progression after treatment cessation in this special population.

## Data availability statement

The original contributions presented in the study are included in the article/**Supplementary Material**. Further inquiries can be directed to the corresponding author. .

## Ethics statement

The study was reviewed and approved by the ethics committee of Second Affiliated Hospital of Chongqing Medical University. Written informed consent was obtained from the individual(s) for the publication of any potentially identifiable images or data included in this article.

## Author contributions

Study concept and design: C-RW, PH; Acquisition of data: C-RW, QZ, X-QL, Y-QD, QT; Analysis and interpretation of data: C-RW, HL, YC, J-SW; Drafting of the manuscript: C-RW; Critical revision of the manuscript for important intellectual content: PH, G-CZ; Statistical analysis: C-RW, G-CZ. Administrative and material support: PH. All authors contributed to the article and approved the submitted version.

## Supplementary material

Supplementary Table 1 Characteristics of biomarkers of 76 analyzed pregnant CHB carriers with HBeAg positive from the TDF and LDT treated group.

Supplementary Table 2 Prediction of HBsAg reduction by HBV biomarker cutoffs.

Supplementary Table 3 Prediction of HBeAg seroconversion by HBV biomarker cutoffs.

Supplementary Figure 1 The flow chart of a process for identifying eligible patients. CHB, Chronic hepatitis B; MTCT, Mother to child transmission; HBsAg reduction, HBsAg decrease >0.3log_10_IU/mL from baseline to last date of follow-up.

Supplementary Figure 2 The changing pattern of HBsAg and ALT levels for each patient with HBsAg reduction (N=13) during follow-up.

Supplementary Figure 3 Correlation of pgRNA, HBcrAg levels and other HBV markers in pregnant CHB carriers with HBeAg positive. A) Correlation matrix of parameters. The size of circle is proportional to Spearman’s correlation coefficient. B) Scatterplots of serum pgRNA or HBcrAg levels (y-axis) vs. other HBV parameters. R= Spearman ‘s correlation coefficient; *P* = *P* value of correlation t-test. Baseline, 24-28 weeks of gestation; near delivery, 32-36 weeks of gestation; postpartum, 2-6 weeks after delivery.

Supplementary Figure 4 Calibration curve for the combined prediction model to predict probability of HBeAg seroconversion.
