# Supplementary material for: PgRNA kinetics predict HBsAg reduction in pregnant chronic hepatitis B carriers after treatment cessation
Source: Front Cell Infect Microbiol. 2022 Dec 12;12:1055774. doi: 10.3389/fcimb.2022.1055774 (PMC9791257; doi:10.3389/fcimb.2022.1055774)
Supplement: Supplementary Table 1 — Characteristics of biomarkers of 76 analyzed pregnant CHB carriers with HBeAg positive from the TDF and LDT treated group. [file Table_1.docx]

| **Table S1. Characteristics of biomarkers of 76 analyzed pregnant CHB carriers with HBeAg positive from the TDF and LDT treated group.** | | | |
| --- | --- | --- | --- |
| Parameter | TDF (N=33) | LDT (N=43) | *P* value |
|  |  |  |  |
| **Baseline** |  |  |  |
| Age, years | 28.0(26.0-31.0) | 28.0(26.0-31.0) | 0.87 |
| Parity status |  |  | 0.43 |
| The first pregnancy | 30 (90.9%) | 41 (95.3%) |  |
| The second pregnancy | 3 (9.1%) | 2 (4.7%) |  |
| Infant's gender, Male, n (%) | 14 (42.4%) | 20 (46.5%) | 0.72 |
| HBeAg seroconversion | 3 (9.1%) | 5 (11.6%) | 0.72 |
| HBsAg reduction | 4 (12.1%) | 9 (20.9%) | 0.31 |
| HBV DNA, log_10_IU/mL | 7.0(6.8-7.4) | 6.7(6.5-7.3) | 0.65 |
| PgRNA, log_10_copies/mL | 8.6(8.4-8.7) | 8.6(8.3-8.7) | 0.19 |
| HBcrAg, log_10_U/mL | 7.9(7.7-8.1) | 7.8(7.6-8.1) | 0.15 |
| ALT, U/L | 24.0(17.0-34.0) | 21.0(15.5-31.0) | 0.73 |
| HBsAg, log_10_IU/mL | 4.5(4.3-4.6) | 4.5(4.2-4.7) | 0.44 |
| HBeAg, log_10_PEIU/mL | 3.2(3.0-3.3) | 3.2(2.9-3.3) | 0.09 |
| **Near delivery** |  |  |  |
| HBV DNA, log_10_IU/mL | 3.2(3.0-4.1) | 3.5(3.0-4.2) | 0.27 |
| PgRNA, log_10_copies/mL | 7.6(7.1-8.0) | 7.7(7.1-7.9) | 0.85 |
| HBcrAg, log_10_U/mL | 8.4(8.2-8.7) | 8.5(8.2-8.6) | 0.52 |
| ALT, U/L | 19.0(16.0-30.0) | 19.0(13.0-25.0) | 0.69 |
| HBsAg, log_10_IU/mL | 4.4(4.1-4.4) | 4.4(3.9-4.6) | 0.89 |
| HBeAg, log_10_PEIU/mL | 3.0(2.8-3.2) | 3.1(2.7-3.3) | 0.98 |
| **Postpartum** |  |  |  |
| HBV DNA, log_10_IU/mL | 5.8(3.0-6.8) | 6.1(4.1-7.0) | 0.16 |
| PgRNA, log_10_copies/mL | 7.7(7.2-7.9) | 7.7(7.1-8.0) | 0.85 |
| HBcrAg, log_10_U/mL | 8.5(8.2-8.6) | 8.4(8.2-8.6) | 0.31 |
| ALT, U/L | 40.0(26.0-57.0) | 35.0(27.0-55.5) | 0.18 |
| HBsAg, log_10_IU/mL | 4.6(4.3-4.7) | 4.6(4.3-4.8) | 0.70 |
| HBeAg, log_10_PEIU/mL | 3.0(2.6-3.2) | 3.1(2.8-3.3) | 0.69 |
| Postpartum ALT _max_ | 58.0 (28.0-88.0) | 42.0 (31.0-67.0) | 0.31 |

Continuous variables were expressed as median [interquartile range (IQR)], and categorical variables were expressed as counts (percentage).

Baseline, at 24-28 weeks of gestation; Near delivery, at 32-36 weeks of gestation; Postpartum, at 2-6 weeks after delivery; TDF, tenofovir disoproxil fumarate; LDT, telbivudine; HBV, hepatitis B virus; DNA, deoxyribonucleic acid; ALT, alanine aminotransferase; HBsAg, hepatitis B surface antigen; HBeAg, hepatitis B e antigen; pgRNA, pregenomic RNA; HBcrAg, hepatitis B core-related antigen. HBsAg reduction, HBsAg decrease by half, namely>0.3log10IU/mL from baseline to last date of follow-up. Postpartum ALT _max_ means peak ALT level postpartum.
