# Supplementary material for: PgRNA kinetics predict HBsAg reduction in pregnant chronic hepatitis B carriers after treatment cessation
Source: Front Cell Infect Microbiol. 2022 Dec 12;12:1055774. doi: 10.3389/fcimb.2022.1055774 (PMC9791257; doi:10.3389/fcimb.2022.1055774)
Supplement: Supplementary Table 2 — Prediction of HBsAg reduction by HBV biomarker cutoffs. [file Table_2.docx]

| **Table S2. Prediction of HBsAg reduction by HBV biomarker cutoffs.** | | | | | | | |
| --- | --- | --- | --- | --- | --- | --- | --- |
| **Variable** | AUC | P value | Specificity | Sensitivity | PPV | NPV | Cut off |
| **Postpartum ALT _max_** | 0.70 | 0.01 | 50.79 | 84.62 | 0.26 | 0.94 | 40.00 |
| **ΔPgRNA log_10_copies/mL** | 0.72 | 0.02 | 76.19 | 69.23 | 0.37 | 0.92 | 0.50 |
| **Combined biomarkers^*^** | 0.79 | ＜0.001 | 65.08 | 84.62 | 0.33 | 0.95 | 0.14 |
| ΔpgRNA, pgRNA decline from baseline to postpartum; Postpartum ALT _max_ means peak ALT level postpartum.; PPV, positive predictive value; NPV, negative predictive value; combined biomarkers^*^ means variable consisting of ΔpgRNA and postpartum ALT _max_. | | | | | | | |
