# Supplementary material for: PgRNA kinetics predict HBsAg reduction in pregnant chronic hepatitis B carriers after treatment cessation
Source: Front Cell Infect Microbiol. 2022 Dec 12;12:1055774. doi: 10.3389/fcimb.2022.1055774 (PMC9791257; doi:10.3389/fcimb.2022.1055774)
Supplement: Supplementary Table 3 — Prediction of HBeAg seroconversion by HBV biomarker cutoffs. [file Table_3.docx]

| **Table S3. Prediction of HBeAg seroconversion by HBV biomarker cutoffs.** | | | | | | | |
| --- | --- | --- | --- | --- | --- | --- | --- |
| **Variable** | AUC | P value | Specificity | Sensitivity | PPV | NPV | Cut off |
| **ALT at baseline** | 0.95 | ＜0.001 | 87.50 | 77.94 | 0.32 | 0.92 | 30.00 |
| **ΔHBcrAg** | 0.83 | 0.01 | 87.50 | 80.88 | 0.35 | 0.98 | 0.35 |
| **Combined biomarkers^*^** | 0.99 | ＜0.001 | 87.50 | 94.14 | 0.63 | 0.98 | 0.09 |

ΔHBcrAg, HBcrAg decline from baseline to postpartum; PPV, positive predictive value; NPV, negative predictive value; combined biomarkers^*^ means variable consisting of ALT at baseline and ΔHBcrAg.
