# Supplementary figures and images for: PgRNA kinetics predict HBsAg reduction in pregnant chronic hepatitis B carriers after treatment cessation
Source: Front Cell Infect Microbiol. 2022 Dec 12;12:1055774. doi: 10.3389/fcimb.2022.1055774 (PMC9791257; doi:10.3389/fcimb.2022.1055774)

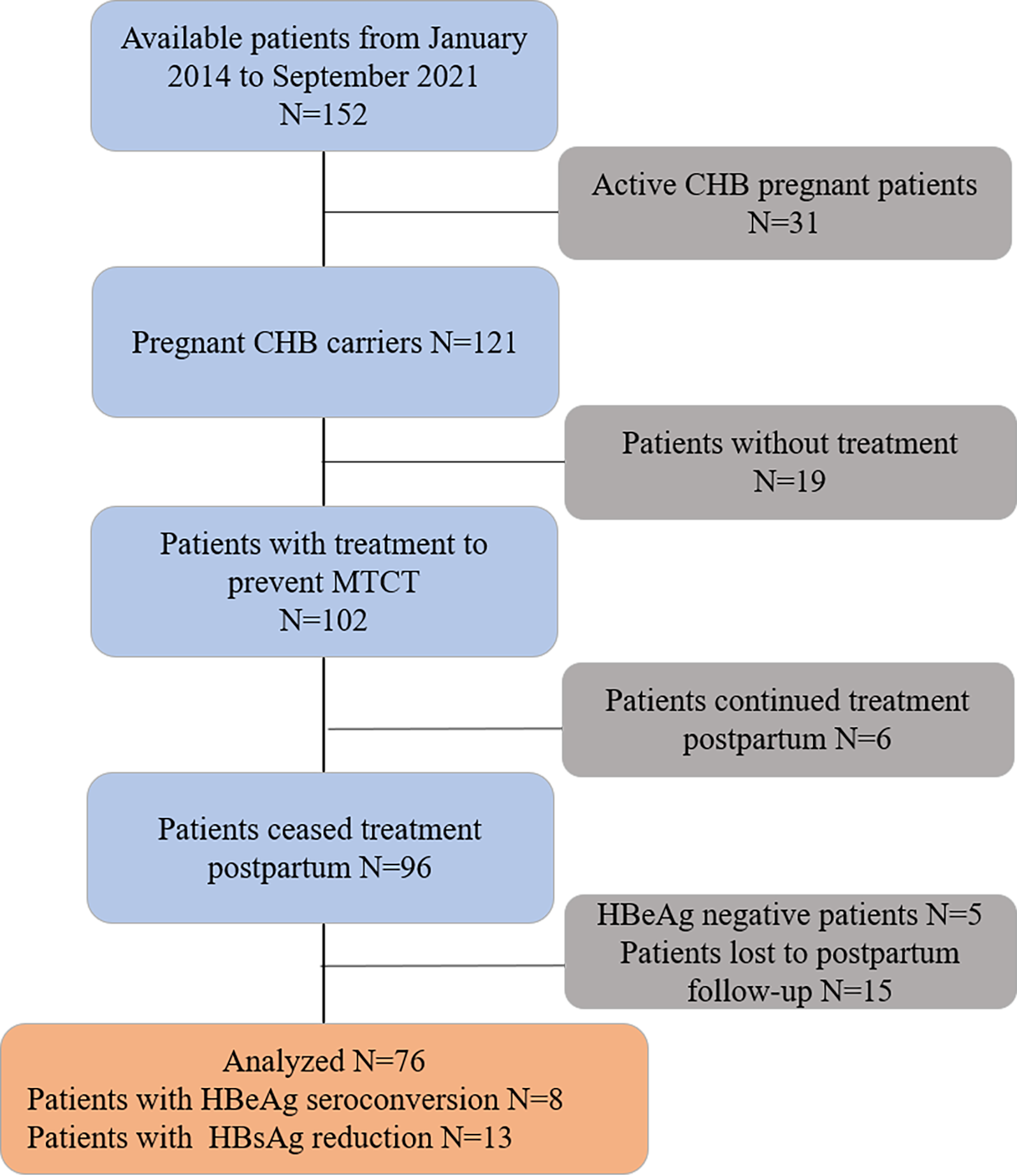

Supplement: Supplementary Figure 1 — The flow chart of a process for identifying eligible patients. CHB, Chronic hepatitis B; MTCT, Mother to child transmission; HBsAg reduction, HBsAg decrease >0.3log10IU/mL from baseline to last date of follow-up. [file Image_1.tif]

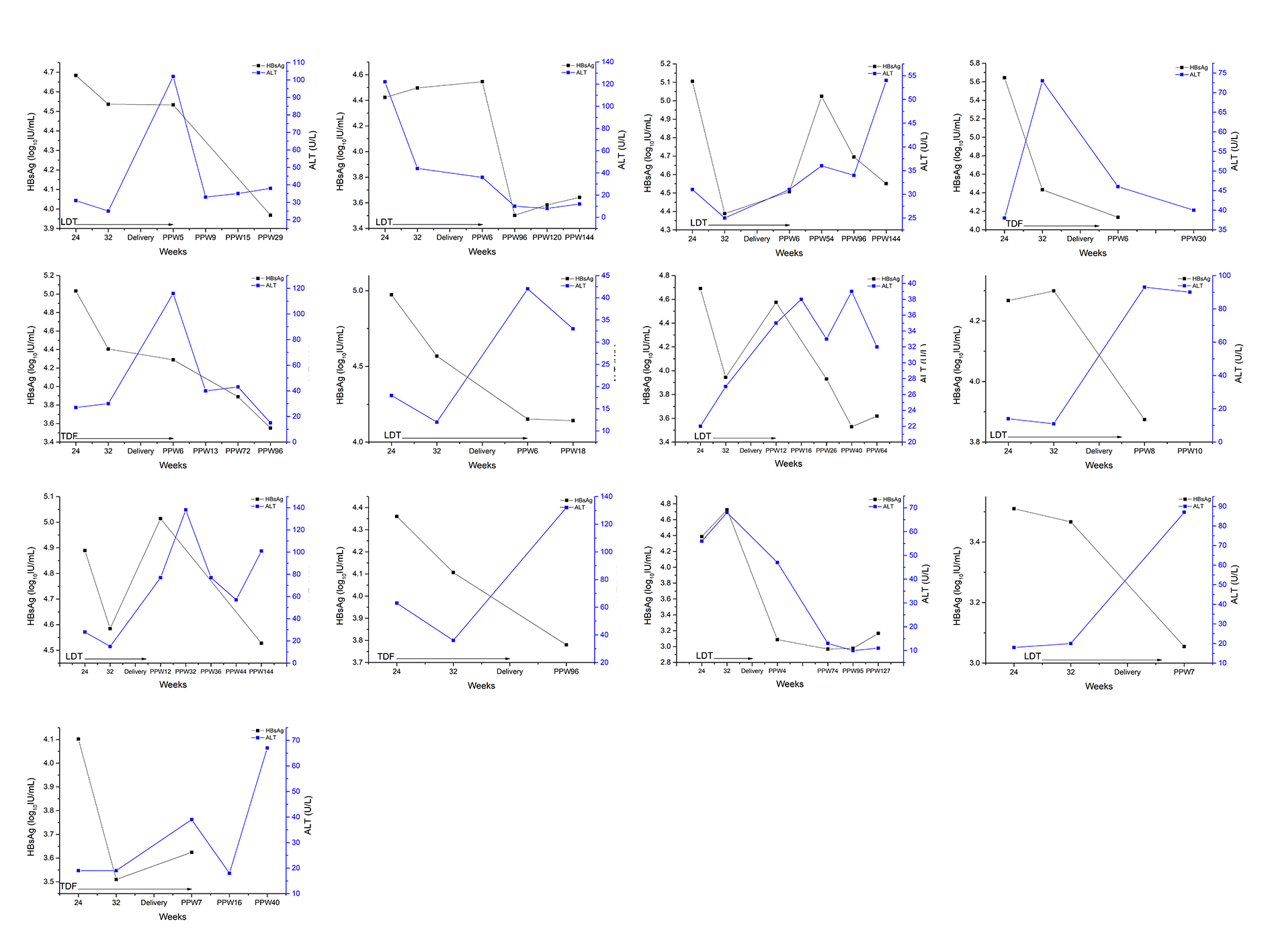

Supplement: Supplementary Figure 2 — The changing pattern of HBsAg and ALT levels for each patient with HBsAg reduction (N=13) during follow-up. [file Image_2.tif]

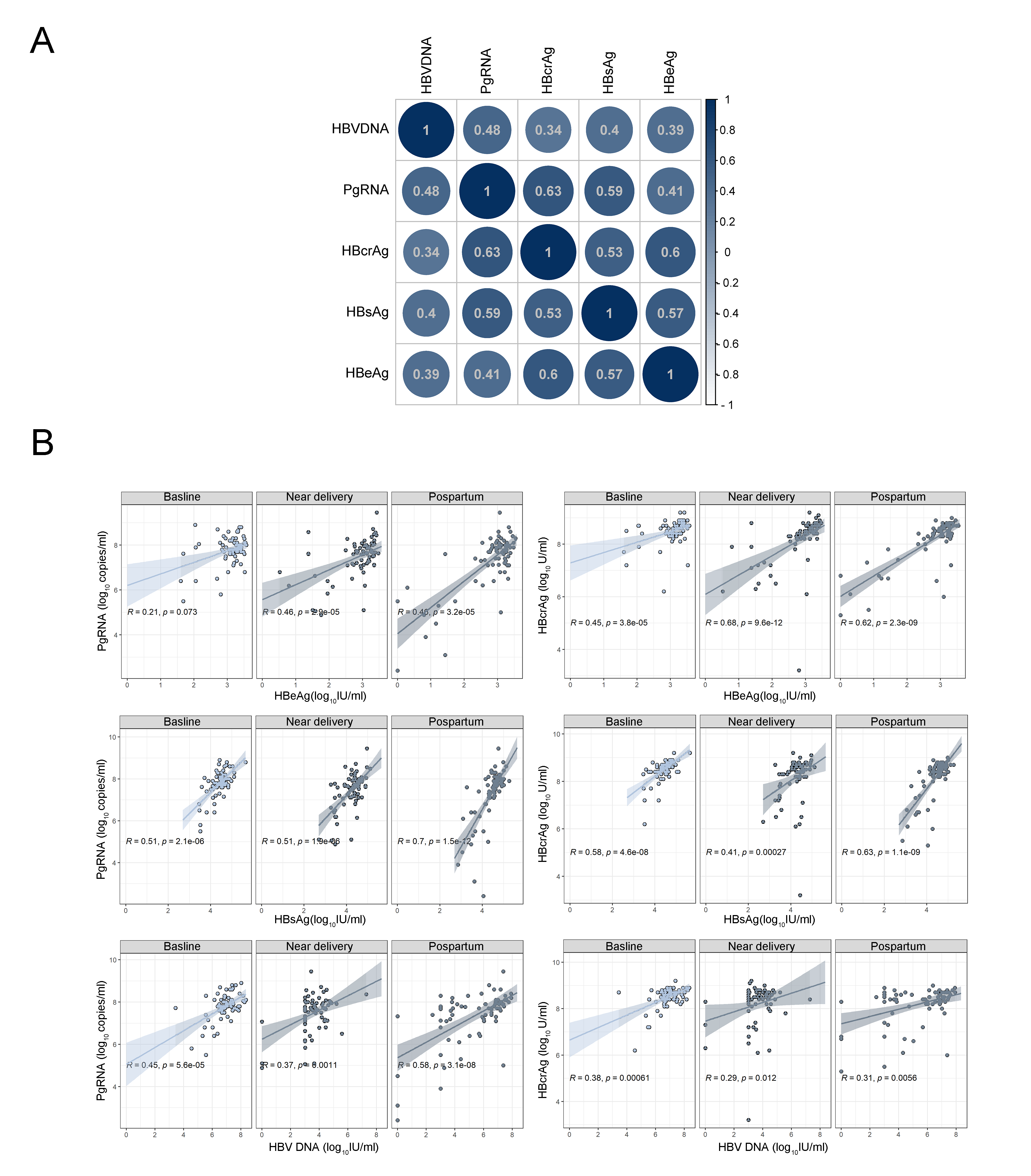

Supplement: Supplementary Figure 3 — Correlation of pgRNA, HBcrAg levels and other HBV markers in pregnant CHB carriers with HBeAg positive. A) Correlation matrix of parameters. The size of circle is proportional to Spearman’s correlation coefficient. B) Scatterplots of serum pgRNA or HBcrAg levels (y-axis) vs. other HBV parameters. R= Spearman ‘s correlation coefficient; P = P value of correlation t-test. Baseline, 24-28 weeks of gestation; near delivery, 32-36 weeks of gestation; postpartum, 2-6 weeks after delivery. [file Image_3.tif]

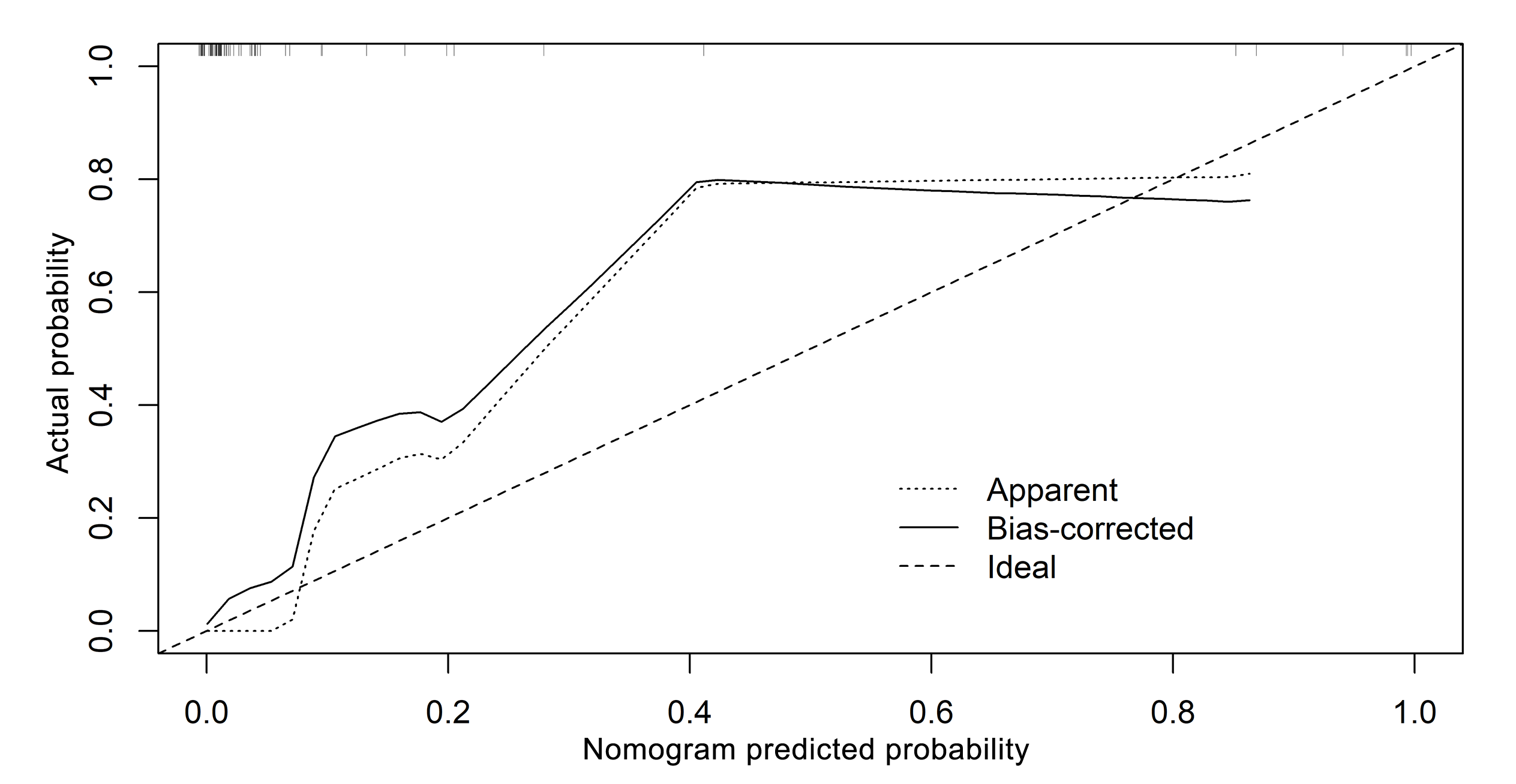

Supplement: Supplementary Figure 4 — Calibration curve for the combined prediction model to predict probability of HBeAg seroconversion. [file Image_4.tif]
